# Evaluation of surrogate tissues as indicators of drug activity in a melanoma skin model

**DOI:** 10.1002/cam4.726

**Published:** 2016-06-23

**Authors:** Palak R. Parekh, Rohini Choudhuri, Urbain Weyemi, Olga A. Martin, William M. Bonner, Christophe E. Redon

**Affiliations:** ^1^ Department of Radiation OncologyGreenbaum Cancer CenterSchool of MedicineUniversity of MarylandBaltimoreMaryland; ^2^ Genomic Integrity GroupLaboratory of Molecular PharmacologyNational Cancer InstituteNational Institutes of Health (NIH)BethesdaMaryland; ^3^ University of MarylandCollege ParkMaryland; ^4^ Division of Radiation Oncology and Cancer Imaging and Molecular Radiation Biology LaboratoryPeter MacCallum Cancer Centre and Department of OncologyUniversity of MelbourneMelbourneAustralia

**Keywords:** Apoptosis, drug response, melanoma tissue model, surrogate tissue, *γ*H2 AX

## Abstract

The development of novel cancer treatments is a challenging task, partly because results from model systems often fail to predict drug efficacy in humans, and also tumors are often inaccessible for biochemical analysis, preventing effective monitoring of drug activity in vivo. Utilizing a model system, we evaluated the use of drug‐induced DNA damage in surrogate tissues as indicators of drug efficacy. Samples of a commercially available melanoma skin model (Mattek MLNM‐FT‐A375) containing keratinocyte and fibroblast layers with melanoma nodules were subjected to various chemotherapeutic regimens for one, four, or eight days. At these times they were analyzed for DNA double‐stranded breaks (*γ*H2AX foci) and apoptosis (TUNEL). A wide range of drug responses in both tumor and normal tissues were observed and cataloged. For the melanoma, the most common drug response was apoptosis. The basal keratinocyte layer, which was the most reliable indicator of drug response in the melanoma skin model, responded with *γ*H2AX foci formation that was abrupt and transient. The relationships between tumor and surrogate tissue drug responses are complex, indicating that while surrogate tissue drug responses may be useful clinical tools, careful control of variables such as the timing of sampling may be important in interpreting the results.

## Introduction

Various strategies to make drug development less costly and more efficient are being constantly tried 1, 2. One strategy is to develop model systems that more closely mimics the in vivo tumor environment while another is to develop the ability to predict drug response in patients, earlier during treatment.

For the first strategy, recently developed systems utilizing tumor cells cultured as multicellular aggregates or embedded in noncellular matrices have been shown to more closely mimic human tissues 3, 4, 5, 6, 7, 8. These studies have also identified differences in drug sensitivity among cells cultured in 2D compared to 3D systems 5, 9, 10, results of which support the increased use of 3D models for drug evaluation 10, 11.

A second strategy is to develop surrogate measures of drug efficacy in patients. During clinical trials, tumor shrinkage or stasis 12 is the best indicator of drug efficacy but these may take considerable lengths of time to become apparent, particularly in tumors that are inaccessible. One option being studied is monitoring drug‐induced metabolic alterations in normal tissues, which may be more easily obtained. Monitoring these surrogate tissues could inform decisions early during treatment, whether the drug regimen is effective, so that the treatment can be discontinued or modified 12. Recently “Phase 0” trials are being assessed as a means to determine whether a drug provokes a response in patients 13. The responses are measured utilizing pharmacodynamics markers and surrogate as well as tumor tissues in evaluating the safety and efficacy of novel anticancer agents 14.

**Figure 1:**
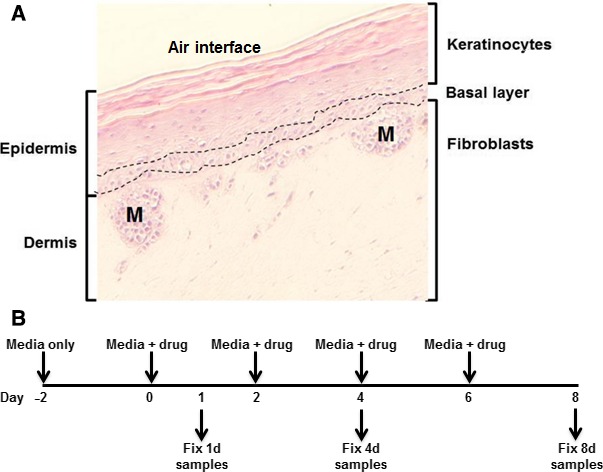
Melanoma skin model (MSM) structure and treatment protocol. (A). H&E section**.** Dashed lines (—) demarcate the keratinocyte basal layer from the differentiating keratinocyte layer above and the fibroblast layer below. Nodules containing A375 malignant melanoma cells can be seen between the basal layer and the fibroblast layer and are noted as “M”. (B). Treatment Protocol**.** Samples were acclimated for 2 days in media alone, then replenished with media + drug every other day. Samples were removed, fixed, and stored on days 1, 4, and 8.

In this study, we utilized a melanoma skin model (hereafter melanoma skin model [MSM]) which consists of melanoma A375 seed tumors located between a stratum of actively dividing basal keratinocytes and differentiated epidermal keratinocytes on one side, and dermal fibroblasts on the other (Fig. 1A). We examined the effects of several genotoxic and non‐genotoxic drugs on the tissues present in the MSM during the 8 days by measuring DNA double‐strand break formation (*γ*H2AX foci formation) and cell death (apoptotic index).

## Materials & Methods

## 3D tissues and anticancer drugs

Melanoma skin model cultures (MLNM‐FT‐A375) and serum free maintenance medium (MLNM‐FT‐MM) were purchased from MatTek, Inc. (Ashland, MA) and maintained according to their protocols. Camptothecin (C9911), temozolomide (T‐2577), Cisplatin (479306), and Gemcitabine (G‐6423) were purchased from Sigma‐Aldrich (St. Louis, MO). Vorinostat (suberoylanilide hydroxamic acid‐SAHA) (10009929) was purchased from Cayman Chemicals (Ann Arbor, MI). Selumetinib (AZD6244) and romidepsin (NSC 630176) were gifts from Dr. Susan Bates (NIH, USA). Drug stock solutions were prepared as per manufacturer's instructions. Stocks for Selumetinib (2.5 mmol/L), camptothecin (20 mmol/L), temozolomide (100 mmol/L), vorinostat (50 mmol/L), and romidepsin (0.5 mmol/L) were prepared with DMSO. Cisplatin (3.33 mmol/L) and gemcitabine (33.3 mmol/L) were prepared with distilled water. The aliquots of stock solutions were stored at −20°C until further use.

### Treatment protocols

Upon delivery, MSM cultures were transferred to six‐well plates and incubated for 2 days at 37°C in a 5% CO_2_ incubator to alleviate any shipment‐induced stresses. Samples were then treated with single drugs at several concentrations or vehicle (DMSO or Water) for 1, 4, and 8 days (Fig. 1B). Drug concentration ranges were determined from survival assays of A375 melanoma cell cultures (data not shown). Samples were fixed in 10% normal buffered formalin (NBF) overnight, stored in 70% ethanol until embedded in paraffin, and sectioned onto microscope slides at the NCI‐Frederick Pathology/Histotechnology Laboratory using standard procedures.

### Immunohistochemistry

Sections attached to slides were deparaffinized in xylene and rehydrated with 100%, 95%, 70% ethanol, distilled water, and phosphate‐buffered saline (PBS) for 10 min each and then stored in prechilled 70% ethanol at 4°C overnight. For antigen retrieval, sections were rinsed in PBS for 10 min, placed in PBS‐Tween 20 (0.05%) for 10 min, incubated in 10 mmol/L sodium citrate pH 6.0–0.05% tween 20 for 25 min at 90°C, cooled for 20 min, washed in PBS‐Tween 20 (0.05%) for 10 min, and blocked with 5% bovine serum albumin (BSA) in PBS‐Tween 20 (0.5%)‐Triton X‐100 (0.01%) (PBS‐TT) for 45 min. After a PBS wash for 10 min, sections were incubated with the primary antibodies in 1% BSA‐PBS‐TT for 2 h. All primary antibodies, mouse *γ*‐H2AX (05‐0636, Millipore Inc, Billerica, MA), rabbit cytokeratin 5 (AF138, PRB160‐P, Covance, Chantilly, VA), and rabbit 53BP1 (NB100‐304, Novus Inc., Littleton, CO) were used at 1:500 dilution. After a PBS wash for 10 min, sections were incubated with the secondary antibodies, goat anti‐mouse Alexa fluor 488 and goat anti‐rabbit Alexa fluor 555 (Life technologies, Grand Island, NY) at 1:500 dilution in 1% BSA‐PBS‐TT for 1 h. After a 10 min wash with PBS, sections were incubated for 20 min at 37°C with RNase A (0.5 mg/mL) and propidium iodide (5 *μ*g/mL), mounted with Vectashield (Vector laboratories, Burlingame, CA) and sealed under coverslips with nail polish. For double staining, nuclei were counterstained with DAPI. The sections were imaged using the LSM 710 (Carl Zeiss, Jena, Germany) or the Nikon PCM 2000 confocal microscopes (Nikon, Japan). *γ*‐H2AX formation was quantitated manually in the different cell types (in 100 cells of each type) of the 3D tissues (fibroblasts, basal layer, keratinocytes, melanoma cells) and was normalized by untreated vehicle control. Data were represented as the averaged relative amounts of two independent experiments.

### TUNEL Assay

Apoptosis was detected using a Tdt‐mediated dUTP Nick End Labeling TUNEL assay kit (11 684795910, Roche Applied Biosciences, Indianapolis, IN, USA) following the manufacturer's protocol, modified as described by Thomas et al. 15. After antigen retrieval performed as described above, sections were blocked with 5% BSA for 45 min, subjected to the TUNEL assay, washed three times with PBS, and stained for *γ*‐H2AX as described above. Nuclei were stained with a mounting medium containing DAPI (Vectashield, Vector laboratories, Burlingame, CA). Sections were analyzed using the LSM 710 laser confocal microscope (Carl Zeiss, Jena, Germany). TUNEL‐positive nuclei were scored as apoptotic.

## Results

### Drug responses of tumor and surrogate tissues in MSM samples

Drugs were classified as genotoxic or non‐genotoxic, depending on their primary mode of action. Genotoxic drugs included camptothecin (CPT), a topoisomerase I inhibitor; cisplatin (CSP), a DNA intercalating agent; gemcitabine (GEM), a nucleoside analog; and temozolomide (TMZ), a DNA alkylating agent. Non‐genotoxic drugs included the MEK inhibitor AZD6244 (AZD) and two HDAC inhibitors, vorinostat (VNST) and romidepsin (RMD). Two types of drug responses were measured: relative DNA double‐strand break formation (*γ*H2AX foci formation) and cell death (apoptotic index) in the three tissues, melanoma, keratinocytes, including the basal keratinocyte layer, and fibroblasts. Most of the agents used in this study are genotoxic and cause DNA damage in both normal and cancerous cells. In mammalian cells, hundreds of histone H2AX molecules are phosphorylated (*γ*H2AX) around DNA double‐strand breaks (DSBs) minutes after DNA damage induction. These *γ*H2AX foci can be visualized with the use of a specific antibody 16. Detection of *γ*H2AX foci by microscopy is very sensitive as a single DNA break can be visualized. A microscopy‐based *γ*H2AX assay allows to measure *γ*H2AX foci in different cell types from a same tissue simultaneously. For all these reasons, *γ*H2AX has been widely used in the last decade to monitor both irradiation and cancer drug responses in patients 17. While pharmacodynamics should be preferentially performed in tumor biopsies, tumor collection may be problematic and/or unsafe for patients. Since many anticancer drugs also target normal cells, several types of cells or tissues that can be obtained noninvasively (surrogate tissues – blood, skin, hair bulbs, and cheek cells) have been used in the clinic for *γ*H2AX detection to mimic drug responses in tumors 18. Thus, we sought to use a microscopy‐based *γ*H2AX assay to monitor DNA damage induction in both normal cells (surrogate tissues – fibroblasts, basal layers, and keratinocytes) and melanoma tumors of the MSM.

**Figure 2:**
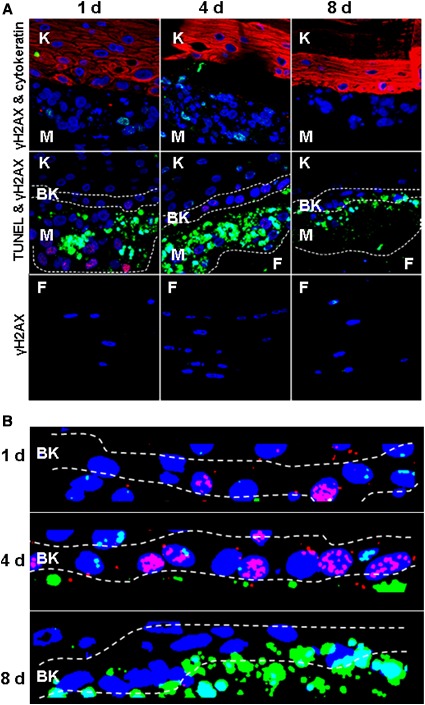
Representative sections of Melanoma skin model (MSM) samples treated with 30 nmol/L camptothecin (CPT), a genotoxic drug**.** Treatment was performed for 1, 4, or 8 days as described in Figure 1B. (A) After treatment, the sections in the row labeled “γH2AX & cytokeratin” were stained for γH2AX (green), DNA (blue), and cytokeratin‐5 (red, a specific keratinocyte marker). Those in the row labeled “TUNEL & γH2AX” were stained for γH2AX (red), DNA (blue), and apoptosis/DNA breaks (green). Those in the row labeled “γH2AX” were of the fibroblast region stained for γH2AX (green) and DNA (blue). (B) Enlargement and enhancement of the basal keratinocyte layer images in panel A (dotted lines) showing transient increase in γH2AX foci (red) during CPT treatment.

In the absence of drug treatment, the melanoma in the seed nodules exhibited both spontaneous apoptosis and *γ*H2AX formation. This observation is in agreement with genomic instability observed in melanoma (Fig. 2) 19. Among the normal tissues, the basal keratinocytes were the most responsive to drug treatments, followed by the fibroblasts. The differentiated keratinocytes were the least responsive. Thus, we found that the most useful comparison of drug responses was between the melanoma and basal keratinocytes.

### Genotoxic drugs

**Figure 3:**
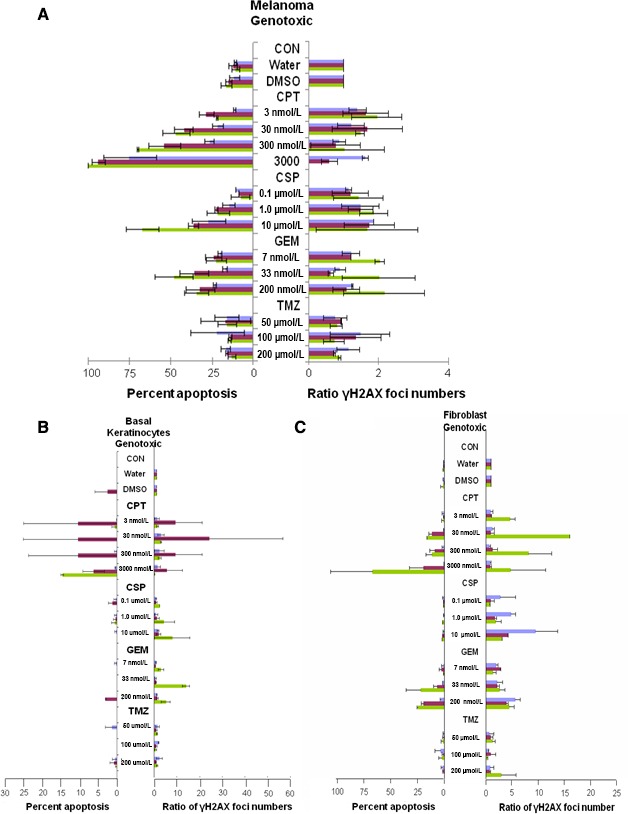
Melanoma skin model (MSM) responses to genotoxic drugs**.** Treated MSM samples like those shown in Figure 2 were analyzed for the extent of apoptosis and γH2AX formation in the melanoma (A), basal keratinocyte (B), and fibroblast (C) layers. Values for % apoptosis are the averaged fraction (%) of apoptotic nuclei in the melanoma after treatment with the noted drug dosages (CPT, CSP, GEM, and TMZ) for 1 day (violet), 4 days (maroon), and 8 days (green). Values for γH2AX formation are the averaged relative amounts compared to vehicle controls from day 0. Error bars represent the standard deviation of two independent experiments performed with different samples several weeks apart. CPT, camptothecin; CSP, cisplatin

Detailed images from MSM samples treated with 30 nmol/L CPT are shown in Figure 2, while results from other genotoxic drugs are shown in Figures S1 & S2. Quantitation of apoptosis and *γ*H2AX foci levels in the three tissues are shown in Figure 3A‐C.

Image analysis of samples treated with 30 nmol/L CPT illustrate that the melanoma and basal keratinocytes exhibited very different responses. The primary melanoma response was apoptosis (Figs. 2A and 3A), reaching 100% at day 8 with 3000 nmol/L CPT, while melanoma *γ*H2AX levels remained near control values with all doses of CPT. Our observations may be related to the fact that during the late apoptotic stage and nuclear fragmentation, *γ*‐H2AX foci formation may rapidly decrease and its use as a marker for apoptosis becomes inappropriate 20, 21. Only a small fraction of cells with subG1 DNA content and being positive for TUNEL may show increased *γ*H2AX levels 21.

In contrast, the basal keratinocytes exhibit substantial but transient increases in *γ*H2AX foci levels, peaking on day 4 and returning to lower levels by day 8 (Figs. 2B and 3B). Fibroblasts also exhibited a large elevation of *γ*H2AX foci levels, reaching a maximum on day 8 (Fig. 3C). Since this is the end of the time course, it is not known whether *γ*H2AX foci levels might continue to increase or decrease as it was observed with the keratinocytes. The abrupt changes in keratinocyte and fibroblast responses are unlikely to be due to fluctuations in the drug concentration since it occurred at all doses of CPT which was replenished every other day. These observations also suggest that timing for tissue collection may be an important consideration in surrogate sample acquisition (late collection may result in increased apoptosis and low *γ*H2AX signal). Thus, *γ*H2AX and TUNEL both collectively can be used for surrogate measurements. Figure 3B & C show that the collective response of *γ*H2AX and apoptosis in normal tissues is correlative with an apoptotic (tumor shrinkage) response in melanoma (Fig. 3A).

For CSP treatment, the results were similar to those obtained with CPT displaying progressive increases in the melanoma apoptotic index with treatment duration and drug concentrations up to 60% with 10 *μ*mol/L at day 8 (Fig. 3A and Fig. S1), while *γ*H2AX foci levels did not differ significantly from the control values. The basal keratinocyte layers treated with CSP exhibited abrupt increases in *γ*H2AX foci levels on day 8 at all doses (Fig. 3B and Fig. S1). Since the increase is at the end of the time course, it is not possible to ascertain whether this change is transient or not. The foci levels in the fibroblast were highest on day 1 rather greatly decreasing thereafter (Fig. 3C). Also, contrary to CPT, there was no real increase of the apoptotic indices for the cells of the keratinocyte and fibroblast layers (Fig. S1 and S3). These findings suggest that normal tissues may have protective responses that enable them to recover after a transient response to continuous drug exposure.

With GEM treatment, the melanoma exhibited moderate increases in apoptotic index reaching 50% with 33 nmol/L GEM after 8 days (Fig. 3A and Fig. S2). In contrast, the basal keratinocytes exhibited increases in *γ*H2AX levels up to 15‐fold after 8 days at 33 nmol/L GEM (Fig. 3B), but no significant apoptosis. The fibroblast layer exhibited several fold increases in *γ*H2AX levels and increased levels of apoptosis at higher drug concentrations and longer exposures (~25% with 200 nmol/L or 33 nmol/L at day 8 and 22% with 200 nmol/L at day 4 vs. ~ 2% of apoptosis for control), results that suggested that GEM was toxic to fibroblasts at the higher doses.

TMZ did not induce any significant changes in either *γ*H2AX levels or the apoptotic indices in the melanoma cells. Similarly, no significant change was observed in these indicators in the basal keratinocyte or fibroblast response to treatment (Fig. 3B and C). Thus, TMZ appeared to have little if any toxicity to the melanoma skin model. Also the lack of response in the keratinocyte and fibroblast layers paralleled the lack of response of the melanoma, indicating these surrogate tissues were accurate indicators.

### Non‐genotoxic drugs

**Figure 4:**
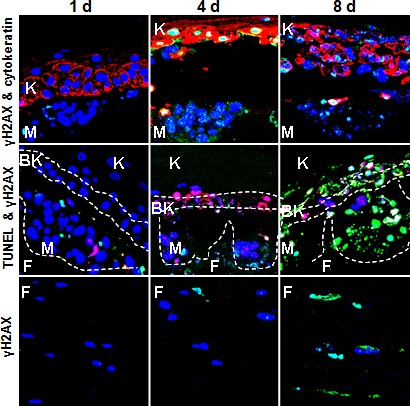
Representative sections of Melanoma skin model (MSM) samples treated with 10 nmol/L RMD, a non‐genotoxic drug. Treatment was performed for 1, 4, or 8 days as described in Figure 1B. After treatment, the sections in the rows labeled “γH2AX” were stained for γH2AX (green), DNA (blue), and cytokeratin‐5 (red, a specific keratinocyte marker). Those shown in the rows labeled “TUNEL” were stained for γH2AX (red), DNA (blue), and apoptosis/DNA breaks (green).

**Figure 5:**
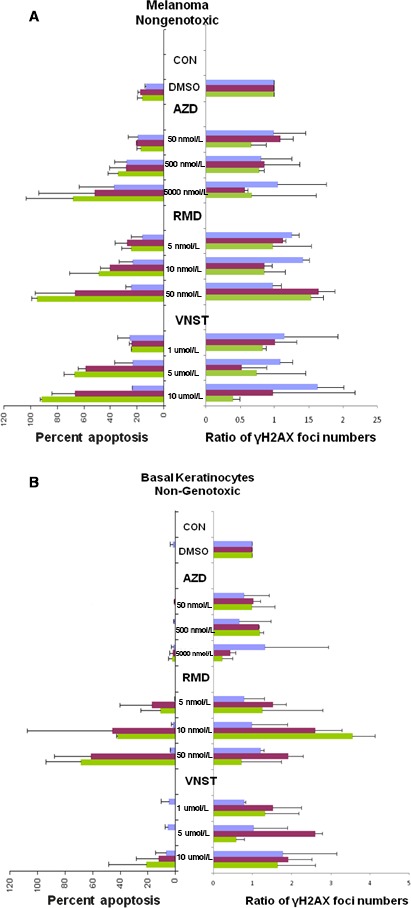
Melanoma skin model (MSM) responses to non‐genotoxic drugs**.** Sections of treated MSM samples like those shown in Figure 4 were analyzed for the extent of apoptosis and γH2AX formation in the melanoma (A) and the (B) basal keratinocyte layer. Values for % apoptosis are the averaged fraction (%) of apoptotic nuclei in the melanoma after treatment with the noted drug dosages (AZD, RMD, and VNST) for 1 day (violet), 4 days (maroon), and 8 days (green). Values for γH2AX formation are averaged relative amounts compared to vehicle controls from day 0. Error bars represent the standard deviation of two independent experiments performed with different samples several weeks apart.

Many chemotherapeutic agents damage cells by means other than inducing DNA damage directly. We examined three representatives of this class, AZD6244 also known as selumetinib (AZD), a selective inhibitor that blocks the activity of MEK (a protein kinase that is part of the key RAS‐RAF‐MEK‐ERK pathway that promotes cell division and survival) and two histone deacetylase inhibitors, vorinostat (VNST) and romidepsin (RMD) that are already FDA‐approved for treatment of cutaneous T‐cell lymphoma 22. Data for the non‐genotoxic drugs are presented in Figures 4 and 5 and Figures S4 and S5.

RMD was found to strongly induce apoptosis in the melanoma reaching 55% at day 4 at 10 nmol/L (Fig. 4 and 5A) with little if any effect on *γ*H2AX foci levels. However, RMD was also toxic to the keratinocytes and dermal fibroblasts, inducing them to undergo apoptosis (Fig. 4, bottom row and 5B, and Fig. S5).

VNST was also found to strongly induce apoptosis in the melanoma reaching 55% to 95% at day 4 and day 8 at the highest dose (Fig. 5 and Fig. S4). However, in contrast to RMD, VNST induced much less apoptosis in the keratinocytes (Fig. 5 and Fig. S4), with no significant increase in *γ*H2AX foci levels.

While AZD treatment increased apoptosis with increasing dose and duration in the melanoma up to about 75% after 8 days at the highest dose (Fig. 5A and Fig. S4), it did not appear to have any effect on the basal keratinocytes at any dosage (Fig. 5B and Fig. S4). This may be due to the mechanism of action of AZD, which is not directly involved in DNA damage but targets cell proliferation. While arresting proliferation could induce apoptosis in melanoma cells which lack various checkpoints, normal cells may arrest in the presence of AZD with much less damage.

## Discussion

This study utilized the melanoma skin tissue model to examine whether normal tissues could serve as surrogates for predicting melanoma responses to a variety of drugs as well as a possible means for vetting novel drugs. The study pinpoints one important aspect in utilizing surrogate tissues to monitor drug effectiveness—that because of the abrupt and transient nature of their responses, the timing of sampling may affect the interpretation of the results.

There is usually a good correlation between drug doses and *γ*H2AX formation in vitro 23, 24. However, we observed different *γ*H2AX/apoptosis kinetics between fibroblasts, keratinocytes, and melanoma in the MSM that suggests a differential DNA damage response that is cell‐type dependent. Similar findings were reported in prostate tissues after irradiation or CPT treatment with a fast and transient *γ*H2AX formation in basal cells and slow and moderate *γ*H2AX response in luminal cells, differences that were linked to constitutively dissimilar responses to DNA damage in the two different cell types 25, 26. It is also known that fibroblasts are more sensitive to UV radiation than keratinocytes, a discrepancy that can be explained by a lower induction of both DNA damage/apoptosis and rapid decrease of p53 in keratinocytes when compared to fibroblasts (fibroblasts showing late and long‐lasting p53 accumulation and were refractory to apoptosis) 27.

**Figure 6:**
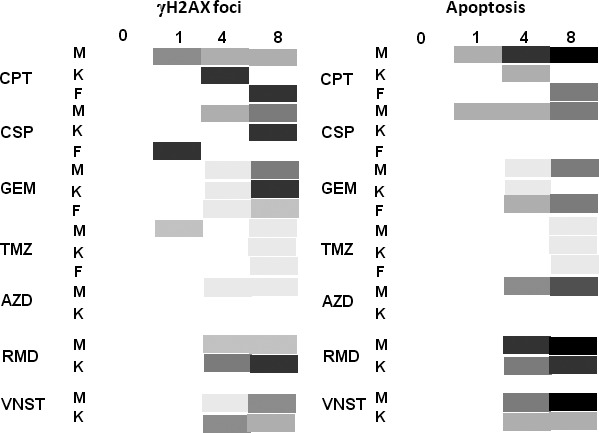
Summary of responses of Melanoma skin model (MSM) tissues to the various drugs in this study**.** Diagram representing γH2AX foci (left) and apoptosis (right) levels in the melanoma skin tissue model prior (0) and during drug treatments (1, 4, and 8 days). Boxes with darker shades of gray symbolize increased γH2AX and apoptosis levels, while white boxes symbolize background levels. CPT: camptothecin; CSP: cisplatin; GEM: Gemcitabine; TMZ: Temozolomide; AZD: Selumetinib/AZD6244; RMD: Romidepsin; VSNT: Vorinostat. M: Melanoma; K: Keratinocytes (Basal); F: Fibroblasts.

Figure 6 presents an overall summary of the results obtained in this study. While three of the genotoxic drugs examined in this study, CPT, CSP, and GEM, were active against the melanoma, inducing increased apoptosis with increased length of treatment, both the basal keratinocytes and fibroblasts exhibited abrupt changes in the levels of *γ*H2AX formation in response to these drugs that were either definitely or possibly transient. Two drugs induced definite transient responses: CPT in keratinocytes where maximal *γ*H2AX foci levels occurred on day 4 and CSP in fibroblasts where maximal *γ*H2AX foci levels occurred on day 1. In both these cases, later time points showed a large decrease in these levels. In several cases, abrupt increases in *γ*H2AX levels occurred on day 8, the end of the time course: for CSP and GEM in the basal keratinocytes and CPT in the fibroblasts. Interestingly, all these results were independent of drug dosage over at least a 100‐fold range. Thus, we conclude that the responses in the surrogate tissues may depend on the identity of the tissues, and the nature, but not the concentration of the drug. These observations show that utilizing *γ*H2AX foci levels in the basal keratinocytes or fibroblasts as a surrogate for the melanoma response is possible but needs to be done with caution.

Among the other genotoxic drugs examined, TMZ exhibited low efficacy against melanoma in these studies, causing only marginal increases in apoptosis after 8 days at 200 *μ*mol/L. Interestingly, although TMZ is a standard clinical treatment for melanoma, it exhibits low efficacy, with less than 20% complete or partial responses 28. Thus, our results for TMZ with MSM were generally consistent with those from the clinic. The basal keratinocytes also exhibited low levels of DNA damage, thus being predictive of the small effect of TMZ on the melanoma.

Among the three non‐genotoxic drugs examined, AZD, RMD, and VNST, all exhibited high efficacies against melanoma, virtually eradicating it by 8 days at the highest concentrations used (Fig. 6). However, with these non‐genotoxic drugs, the basal keratinocytes were less useful as a surrogate. AZD and VNST failed to induce significantly elevated *γ*H2AX levels in the keratinocytes. RMD did induce elevated levels of *γ*H2AX but also induced apoptosis in the keratinocytes (Fig. 4 and 5B), indicating it was cytotoxic. Our data clearly indicates that nanomolar range of RMD is sufficient to cause toxicity issues in melanoma tumors containing A375. Similarly, a study by Kobayashi et al., 29 show the HDAC inhibitor FK228/RMD to be very effective in killing malignant melanoma when compared to other drugs. Interestingly, the cytotoxic effect of FK228/RMD was found to be mediated through the upregulation of Rap1, a small GTP‐binding protein of the Ras family, overexpressed in cells carrying the V599E mutation of B‐Raf 29. Rap1 was shown to form a complex with the mutated BRAF, suppressing the activation of Ras‐MAP kinase signaling and leading to apoptosis in malignant melanoma. Interestingly, A375 melanoma cells are carrying a similar mutation, V600E, in the activation loop of BRAF. Thus, RMD as well as other HDAC inhibitors may act as an endogenous regulator of Ras‐MAP kinase signaling in melanomas and may have high effectiveness in killing BRAF‐mutant cells.

While AZD is highly cytotoxic to melanoma from the MSM, the response in patients is varied and seems to be correlated with the BRAF mutation status. In a phase II trial, AZD, in combination with docetaxel, shows no significant improvement in progression‐free survival (PFS) compared to docetaxel alone in patients with wild‐type BRAF 30. In the opposite, improved PFS and tumor regression was observed with AZD when compared to chemotherapy in advanced uveal melanoma patients 31. However, another trial revealed that while no significant difference in PFS was observed between patients with stage III/IV melanoma unselected for BRAF mutations, five of six patients with partial response to AZD had BRAF‐mutant tumors (mostly V600E) 31. A study by Catalanotti et al. 32 confirms these observations as AZD treatment resulted in tumor regression in three of five patients with BRAF‐mutated (V600E or V600K) and low pAKT melanomas 32. Similarly, a better response and longer time to progression were observed with AZD in patients who had BRAF‐mutation tumors compared to patients with wild‐type BRAF 33. These clinical observations may be linked to our observations with the MSM in which AZD lead to accrued cell death of tumors containing the BRAF V600E‐mutant A375 cells.

Data from clinical trials for the use of RMD and VNST against melanoma are very limited. A phase I clinical trial intended to determine the toxicity and tolerability of escalating doses of VNST with doxorubicin showed potential for this regimen in melanoma 34. Another study combining marizomib, a proteasome inhibitor, to VNST resulted in a highly synergistic antitumor activity with stable disease in 61% of patients with 39% having reduction in tumor size 35. VNST also demonstrated a high proportion of patient (50%) with stable disease in a phase II trial. The authors of this study suggested exploring the effect of VNST with BRAF mutation in the future 36.

Overall, of the seven drugs tested, we show that CPT and VNST are the best to selectively kill melanoma cells in a 3D tissue model. These two drugs eradicate tumor cells without inducing significant toxicity in normal cells. While CPT is not currently used because of its poor solubility and stability in the plasma, its derivatives irinotecan and topotecan as well as noncamptothecin topoisomerase I inhibitor are being developed and tested in the clinic (e.g., ClinicalTrials.gov Identifiers NCT01336985, NCT00101270, 37, 38). We show here that TMZ is the less effective drug to kill melanoma in 3D tissue. TMZ is one of the best drug candidate to treat melanoma, and phase I and II trials of TMZ have shown response rates ranging from 0% to 29%, with complete responses observed in 0 to 17% of patients 39. A report by Carvajal et al. 40 showed TMZ failed to demonstrate an objective response (tumor shrinkage) during melanoma treatment. Prolonged administration schedule of TMZ is associated with an unusual, but manageable toxicity 41. This latter observation can be related to the fact that TMZ have a low genotoxic impact on normal tissue in MSM.

An increasing number of clinical trials are testing drug combination for melanoma. The motivation for combination chemotherapy is to use drugs that work by different mechanisms or target specific tumor mutation 42. For this reason, the 3D tissue model may be a valuable tool to help develop new drug combinations.

In conclusion, we think that our findings are important for several reasons. First, this study is novel as it is the first time a melanoma 3D skin model is used to evaluate a large array of anticancer drugs with different signal pathways. Second, some of the endpoints observed in the 3D tissue can be correlated with observations made in the clinic. Third, relative drug responses between normal tissues and tumor tissues may give clues on drug tolerance in patients. However, while our studies indicate that MSM and similar systems may serve as a preclinical aid to develop novel drugs and test surrogates to predict tumor response, they also show that considerable caution is needed in interpreting the results. Further studies may help establish the utility of 3D tissues for drug development.

## Conflict of Interest

None declared.

## Supporting information


**Figure S1.** Representative sections of MSM samples treated with genotoxic drugs. Treatment was performed with various concentrations of camptothecin (CPT) (left panel), or temozolomide (TMZ) and cis‐platinum (CSP) (right panel) for 1, 4, or 8 days as described in Figure 1B. After treatment, the sections in the rows labeled “*γ*H2AX” were stained for *γ*H2AX (green), DNA (blue), and cytokeratin‐5 (red, a specific keratinocyte marker). Those shown in the rows labeled “TUNEL” were stained for *γ*H2AX (red), DNA (blue), and apoptosis/DNA breaks (green). Representative sections are shown. Insets are enlarged regions of the images to illustrate the types of structures measured, those at the top for the basal and keratinocyte layers, those at the bottom for the melanoma regions. Magnification was 100x for the images and 300x for the insets.

**Figure S2.** Representative sections of MSM samples treated with GEM. Treatment was performed with various concentrations of GEM for 1, 4, or 8 days as described in Figure 1B. After treatment, the sections were stained for *γ*H2AX (green), DNA (blue), and cytokeratin‐5 (red, a specific keratinocyte marker). Representative sections are shown. Magnification was 100x.

**Figure S3.** Representative fibroblast sections treated with CPT and CSP. Treatment was performed with as noted for 1, 4, or 8 days as described in Figure 1B. After treatment, the sections were stained for *γ*H2AX (green) and DNA (blue). Representative sections are shown. Magnification was 100x.

**Figure S4.** Representative sections of MSM samples treated with non‐genotoxic drugs. Samples of MSM were treated with various concentrations of the non‐genotoxic drugs, 5‐azacytidine AZD6244 (AZD) (left panel), vorinostat (VNST) or romidepsin (RMD) (right panel) for 1, 4, or 8 days as described in Methods. The DMSO images represent untreated controls. See Figure S1 for details.

**Figure S5.** Representative fibroblast sections treated with AZD and RMD. Treatment was performed with as noted for 1, 4 and 8 days as described in Figure 1B. After treatment the sections were stained for *γ*H2AX (green), DNA (blue), and cytokeratin‐5 (red, a specific keratinocyte marker). Representative sections are shown. Magnification was 100x.
