# Supplementary material for: Evaluation of surrogate tissues as indicators of drug activity in a melanoma skin model
Source: Cancer Med. 2016 Jun 23;5(8):1731–41. doi: 10.1002/cam4.726 (PMC4971901; doi:10.1002/cam4.726)
Supplement: Supplementary file 6 [file CAM4-5-1731-s006.docx]

Supplementary Information

Supplementary Figure 1. Representative sections of MSM samples treated with genotoxic drugs. Treatment was performed with various concentrations of camptothecin (CPT) (left panel), or temozolomide (TMZ) and cis-platinum (CSP) (right panel) for 1, 4 or 8 days as described in Figure 1B. After treatment, the sections in the rows labeled "γH2AX" were stained for γH2AX (green), DNA (blue) and cytokeratin-5 (red, a specific keratinocyte marker). Those shown in the rows labeled "TUNEL" were stained for γH2AX (red), DNA (blue) and apoptosis/DNA breaks (green). Representative sections are shown. Insets are enlarged regions of the images to illustrate the types of structures measured, those at the top for the basal and keratinocyte layers, those at the bottom for the melanoma regions. Magnification was 100x for the images and 300x for the insets.

Supplementary Figure 2. Representative sections of MSM samples treated with GEM. Treatment was performed with various concentrations of GEM for 1, 4 or 8 days as described in Figure 1B. After treatment, the sections were stained for γH2AX (green), DNA (blue) and cytokeratin-5 (red, a specific keratinocyte marker). Representative sections are shown. Magnification was 100x.

Supplementary Figure 3. Representative fibroblast sections treated with CPT and CSP. Treatment was performed with as noted for 1, 4 or 8 days as described in Figure 1B. After treatment, the sections were stained for γH2AX (green) and DNA (blue). Representative sections are shown. Magnification was 100x.

Supplementary Figure 4. Representative sections of MSM samples treated with non-genotoxic drugs. Samples of MSM were treated with various concentrations of the non-genotoxic drugs, 5-azacytidine AZD6244 (AZD) (left panel), vorinostat (VNST) or romidopsin (RMD) (right panel) for 1, 4 or 8 days as described in Methods. The DMSO images represent untreated controls. See Supplementary Figure 1 for details.

Supplementary Figure 5. Representative fibroblast sections treated with AZD and RMD. Treatment was performed with as noted for 1, 4 and 8 days as described in Figure 1B. After treatment the sections were stained for γH2AX (green), DNA (blue) and cytokeratin-5 (red, a specific keratinocyte marker). Representative sections are shown. Magnification was 100x.
